# The glass ceiling thickens: the impact of COVID-19 on academic medicine faculty in the United States

**DOI:** 10.1080/10872981.2022.2058314

**Published:** 2022-03-28

**Authors:** Rebecca S. Lufler, Margaret A. McNulty

**Affiliations:** a Department of Medical Education, Tufts University School of Medicine, Boston, MA, USA; b Department of Anatomy, Cell Biology, and Physiology, Indiana University School of Medicine, Indianapolis, IN, USA

**Keywords:** COVID-19, academic medicine faculty, women, childcare, mental health

## Abstract

The inequities faced by women in academic Medicine before the COVID-19 pandemic are well established. However, there is little formal data regarding exactly how the pandemic has affected faculty. This cross-sectional study investigated the impact of the pandemic on responsibilities at home, work, and mental health according to gender identification, faculty rank, and faculty appointment. In February 2021, an online questionnaire was broadly distributed to academic medicine faculty. Respondents were asked to provide demographic data, answer questions about their responsibilities at home and work, mental health, and how the pandemic has influenced these. Respondents were also asked to document what their institution(s) can do to help faculty through the pandemic. Responses were analyzed via Pearson’s chi-square tests and thematic analysis. Women faculty were more likely to be responsible for the care of others (70%, p = 0.014), and the impact was negative, especially for early career faculty (p = 0.019). Productivity in research, teaching, and clinical practice were negatively impacted, with women feeling this in clinical practice (p = 0.005), increased teaching load (p = 0.042), and inadequate work environment (p = 0.013). In the areas of self-care and mental health, women (p < 0.001), early career-faculty (p < 0.001), and clinical faculty (p = 0.029) were more negatively impacted. Early-career women were more likely to fear retribution. Five themes emerged, including Flexible Expectations, Support, Mental Health, Compensation, and Communication. Pre-pandemic stress and burnout were rampant, and this study demonstrates that academic medicine faculty are still suffering. It is the authors’ hope that administrations can utilize these data to make informed decisions regarding policies enacted to assist populations who are most vulnerable to the effects of the pandemic.

## Introduction

Inequities in academic medicine are well documented, as the ‘pyramid problem’ or the ‘leaky pipeline’, the phenomenon by which women occupy fewer positions at each subsequent level of the academic hierarchy, has existed for decades [1]. Representation of women at each rank is thought to decrease due to their likelihood to leave academia because of various factors, such as issues affecting work-family conflict [2,3]. In academic medicine specifically, men occupy a greater percentage of full-time faculty positions, as well as a greater percentage of each rank [4]. The pyramid problem remains as of 2018, with women occupying 58% of instructor, 46% of assistant professor, 37% of associate professor, and 25% of full professor positions [4]. This is exacerbated in leadership, where women make up 30% or less of division and section chiefs, center and institute directors, and department chairs. Some may argue that women have greater representation in administrative leadership positions, occupying 52% of assistant dean positions, 47% of associate dean positions, and 34% of senior associate or vice dean positions, however it should be noted that administration itself is a leaky pipeline, leading to only 18% of medical school deans who are women [4].

The leaks of women out of the pipeline have been attributed in part to the difficulties surrounding having a career and children. Early career women in academia have children at the same rates as men yet receive lower wages; late career women are still paid less, yet are less likely to have children [1,5]. The literature is replete with studies on who bears the brunt of the childcare duties and shows women spend more time caring for children as well as on household duties, creating higher levels of work-family conflict [1,3,5–9]. Implicit and explicit gender bias have prevented women from being chosen for leadership positions and have contributed to harassment in the workplace, thereby creating toxic work environments in academic medicine [6,10,11]. With increased levels of work-family conflict and battling gender bias at work, it is not surprising that women with children experience significantly more stress [2,12,13].

The misconception that there are gender disparities in how many hours faculty work, therefore leading to pay and productivity inequities, has been widely debunked [14]. Data from the AAMC Faculty Salary Survey from 2017 show that median compensation for men was not only greater at every rank, but the gap widened at higher ranks of chief ($87,000 difference) and chair [$105,000 difference; 15]. The gap in median compensation was smaller for women in basic science compared to clinical departments, and within the clinical science departments, smaller for women with PhD degrees. It must be noted that reported work hours per week were similar between both genders, differing by 3 hours or less per week, and these compensation gaps have not changed drastically over the previous five years [15].

In a longitudinal study of academic productivity, no gender differences in acquiring federal grant funding among medical school faculty were found, however, women had a lower rate of publication and h-index, which are imperative metrics in the promotion process [16]. Women are more likely to volunteer for time consuming service duties, such as committee roles and mentoring [17]. All of these factors, in conjunction with greater responsibilities at home, can lead to fewer women being promoted [15].

When it comes to prevalence of burnout, stress, and quality of life, the trends are similar to aforementioned inequities. Burnout has been shown to affect university faculty, regardless of field [18–20], with females and early career faculty experiencing the burnout factor ‘emotional exhaustion’ more than males and late career faculty [21,22]. As the fallout from academic faculty burnout and stress includes depression, substance abuse, and suicidal thoughts, which in turn adversely affects patient care, student education, and mentorship, it is imperative to match strategies to specific causes of burnout [23–25].

As COVID-19 spread and was categorized as a pandemic, faculty in academic medicine in particular were faced with drastic changes to their everyday lives. Clinical faculty became front line healthcare workers. Faculty were forced to quickly adopt virtual methods of teaching and clinical practice. Medical school curricula changed, forcing faculty to work constantly to deliver a safe, yet complete, education to students. Research labs were forced to close, requiring faculty to halt their work and find ways to regain productivity with new safety protocols and fewer research students and staff. Decreases in working hours devoted to research have affected basic scientists and have disproportionately affected women with young dependents at the undergraduate level [26,27]. Articles and opinion pieces have dominated the literature surrounding how the pandemic has affected academic medicine faculty, yet little formal research has been conducted in this area. Many predict it will raise existing barriers to success, leading women to flounder in early career positions or leave the workforce [28–31]. A few early studies have shown that the publication gender gap has persisted throughout the COVID-19 pandemic [32], and has resulted in fewer publications by women and fewer women scientific experts and leaders being quoted in the media on the pandemic itself [33,34]. These publication and media biases have the potential to have long-lasting effects on gender disparities in academic medicine. Knowing that the pre-COVID inequities already existed, some publications have focused on suggestions and strategies to mitigate their exacerbation and help faculty manage work-life balance and achieve career advancement [28,35–39], however it is difficult to make action plans without knowing exactly how faculty are being affected.

There is no question that this extra work on top of the burden of increased responsibilities at home and the impact of the national health crisis on mental health has had extraordinary effects on faculty in academic medicine. However, much of the evidence of the pandemic’s impact has centered around undergraduate institutions [26,27,34,40,41] and opinion pieces [28, 31, 35–39]; however, the pandemic continues to wear on academic medicine faculty. The purpose of this study is to elucidate the impact of the COVID-19 pandemic on faculty in academic medicine, specifically to determine if the pandemic is disproportionately impacting faculty responsibilities at home and work, and their mental health according to gender identification, faculty rank, and faculty appointment.

## Methods

### Participants and questionnaire measures

A questionnaire was distributed to academic medicine faculty as defined by faculty employed by an academic health center/school of medicine located within the USA. The questionnaire was broadly distributed via local institutional email listservs, national association listservs and message boards to which authors are members (e.g., AAMC Council of Faculty and Academic Societies), and social media (Twitter), and was open for response February–March 2021. Participants were invited to complete the anonymous questionnaire via a link to Qualtrics XM online survey software (Qualtrics, Provo, UT). This study was given exempt determination by the Tufts Medical Center/Tufts University Health Sciences Institutional Review Board, IRB# 1268.

The questionnaire was composed of 24 items, including multiple choice, select all that apply, or yes/no (18), open ended (4), and multi-level Likert-scale (2) questions. The Likert-scale questions also provided an opportunity for additional information with an additional open response option. Questions were derived from a review of the literature and faculty experiences on AAMC Council of Faculty and Academic Societies with feedback from the Associate Dean for Faculty Development at Tufts University School of Medicine. The questionnaire asked respondents to answer questions on their background information: institution location, terminal degree, academic rank, tenure track/tenure status, percent effort for academic activities (clinical service, teaching, service, research/scholarship, and other), and race and gender identification. Questions about responsibilities outside of work included descriptions of elder- and childcare, and distribution of care responsibilities before and since the onset of the COVID-19 pandemic. Questions on the impact of the COVID-19 pandemic included overall impact on research, teaching, clinical practice, and specific impact on caring for others, self-care, mental health, racial trauma, household responsibilities, reduced access to space, increased teaching activities, inadequate work environment, work productivity, and patient care. These questions asked respondents to rate the impact of the pandemic on a 5-point Likert scale, significant negative impact, low negative impact, no impact, low positive impact, and significant positive impact. Respondents had the opportunity to indicate if they considered taking a leave of absence or tenure clock extension, if they fear retribution for doing so, whether they have been supported by their institution, and if they feel the pandemic will have a negative impact on job promotion. Finally, respondents were provided an opportunity to document via free text what the academic institution can do to help faculty through these pandemic times.

### Statistical analysis

Data were exported from Qualtrics to Microsoft Excel (version 16.43, Microsoft, Redmond, WA). Incomplete responses (defined as <80% complete) were removed and coded categorical responses to nominal data for subsequent analyses. All remaining data were visually analyzed and tested for normality using the Shapiro–Wilk test. Responses indicating ‘Not Applicable’ for specific parameters (e.g., questions pertaining to the impact of the pandemic on various aspects of one’s work) were removed from the analyses. In order to assess effects of the pandemic on subgroups of faculty, a ‘degree type’ variable was created to divide respondents into those with a clinical degree (e.g., M.D., P.A., D.O., Pharm.D., P.T.) versus a non-clinical degree (e.g., Ph.D., M.P.H). Further, to assess effects of the pandemic on faculty at different career levels, a ‘career level’ variable was developed that combined ‘ranks’ accordingly: Instructor/Lecturer, Assistant Professor, and Clinical Assistant Professor were coded as ‘early career faculty’; Associate Professor and Clinical Associate Professor were coded as ‘mid-career faculty’; and Professor and Clinical Professor were coded as ‘late career faculty’. Outcome variables included impact on research, teaching, clinical practice, and specific impact on caring for others, self-care, mental health, racial trauma, household responsibilities, reduced access to space, increased teaching activities, inadequate work environment, work productivity, and patient care. Descriptive data, specifically, the percent of each response within a given group of respondents, were calculated. Categorical data were analyzed using Pearson’s chi-squared tests to evaluate how likely the observed differences between groups arose by chance. All statistical analyses were computed using SPSS statistical software (IBM® SPSS® Statistics v. 27, Armonk, NY, USA).

Six-step thematic analysis [42] was undertaken to answer the question: ‘What do you think the academic institution can do to help faculty through these pandemic times?’ Two individuals (R.S.L. and M.A.M.) undertook initial coding of narrative responses to this question and developed a codebook whereby responses were taken at face value and not interpreted. Using comparative methods, discrepancies in codes were addressed and a final codebook was developed. To increase the reliability of the results, data triangulation was employed to reach data saturation [43]. Data saturation was reached when new additional information was unable to be obtained and no new codes occurred in the data [44]. Data were managed using NVivo software, version 12 (QSR International, Melbourne, Australia).

### Questionnaire validity and reliability

Exploratory factor analysis (EFA) and Cronbach’s alpha reliability coefficients were utilized as methods of questionnaire item validity and reliability, respectively. The EFA analysis yielded four factors that explained 68% of the variation in the data. These factors included: (1) Care for Others (including: impacts on clinical practice, patient care, and caring for others), (2) Personal Impacts (including: impacts on self-care, mental health, work production, and household responsibilities), (3) Impact on Work (including: impact on work production, inadequate work environments, reduced access to work, and household responsibilities), and (4) Impacts on Teaching (including: impacts of increased teaching and impacts on teaching). Two items did not load in any factors: impact on racial trauma and impacts on research. Additionally, two items, impact on work production and household responsibilities loaded into both Personal Impacts and Impact on Work, however their communalities were below 0.5 and therefore viewed as insufficient. Following EFA analysis, Cronbach’s alpha was relatively high (alpha = 0.773). Additionally, by examining several of the same constructs in both the quantitative and qualitative strands of the study, convergent validity was therefore utilized to strengthen the validity of the findings [45].

## Results

The questionnaire received 325 viable responses. Data reported below outlining the negative impacts on various aspects of work combine ‘low negative impact’ and ‘significant negative impact’ unless otherwise stated.

**Figure 1.**
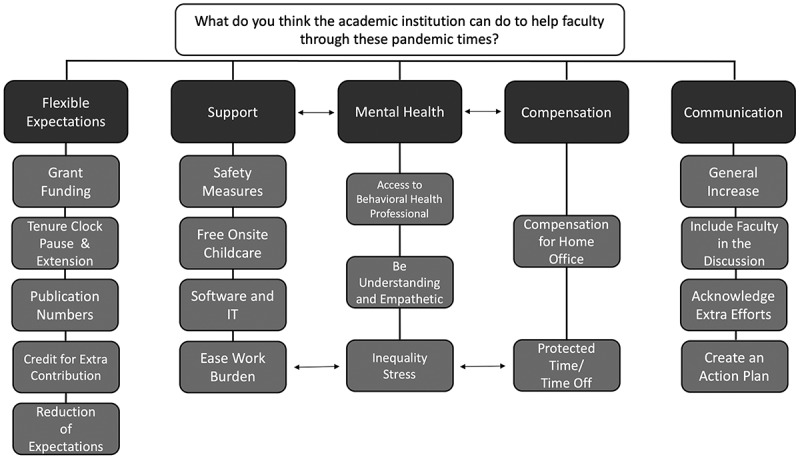
Themes (dark grey boxes) and subthemes (light grey boxes) that emerged from thematic analysis of open-ended responses.

Thematic analysis of the narrative responses from academic medicine faculty on how institutions can help them revealed five themes (Figure 1): **Support, Flexible Expectations, Communication, Compensation, and Mental Health**. *Subthemes* within these themes are highlighted in the below text.

### Demographic data of respondents

**Figure 2.**
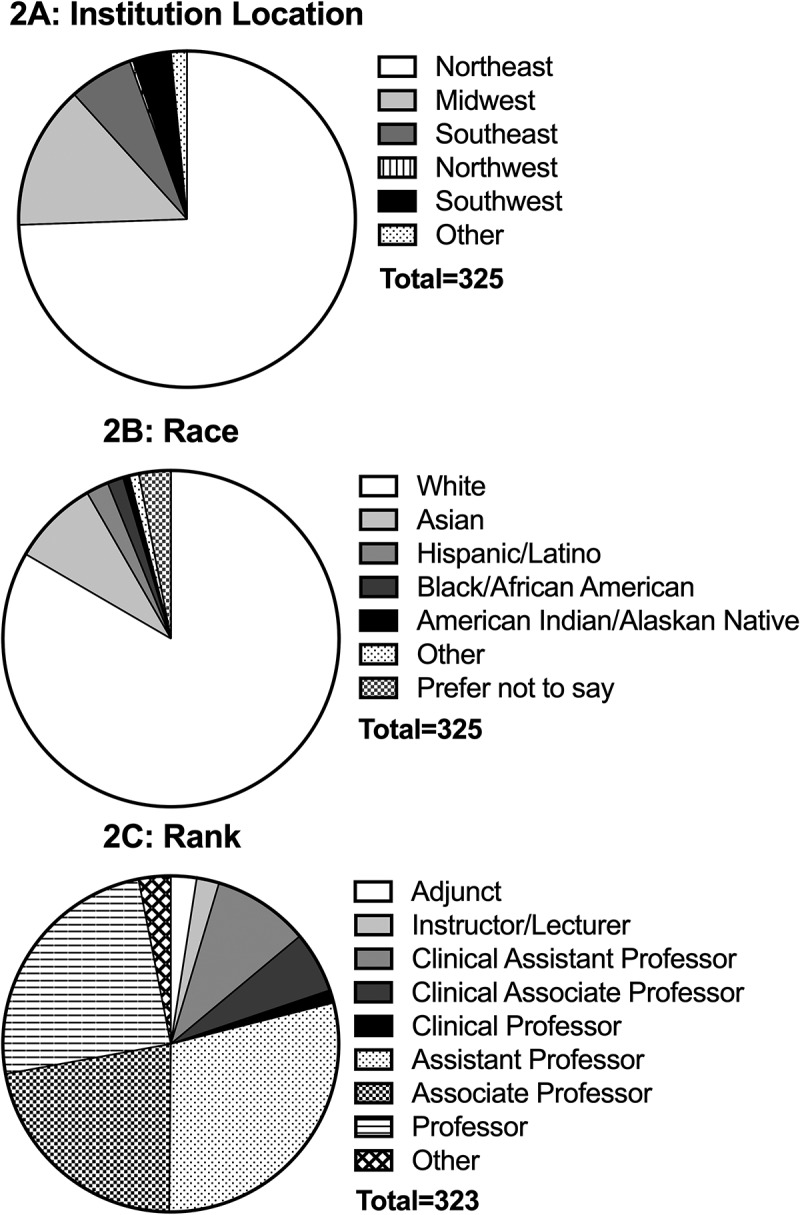
Demographic data of respondents.

All contiguous U.S. regions were represented and the vast majority of respondents (n = 242, 74%) were located in the Northeast (Figure 2(a)). Respondents were split evenly between females (n = 161, 50%) and males (n = 158, 49%), with 6 respondents preferring not to indicate their gender among the five options (female, male, non-binary, transgender female, transgender male). Respondents were predominantly white (n = 271, 83%; Figure 2(b)). The vast majority of respondents held either an M.D. (n = 152, 47%) or Ph.D. (n = 124, 38%); the remaining 49 respondents held either a combination of these degrees (M.D./Ph.D.) or other degrees (e.g., M.P.H., P.A., Pharm.D., D.O., etc.). After creating the ‘degree type’ variable, 179 respondents held a clinical degree (56%) and 139 respondents held a non-clinical degree (44%). Respondents were mostly employed within the Assistant Professor-Associate Professor-Professor line (Figure 2(c)), and the majority were on a tenure-track line (n = 235, 72%).

### Women in early- or mid- career stages were most likely to be responsible for the care of others and report the negative impact on this extra effort

**Figure 3.**
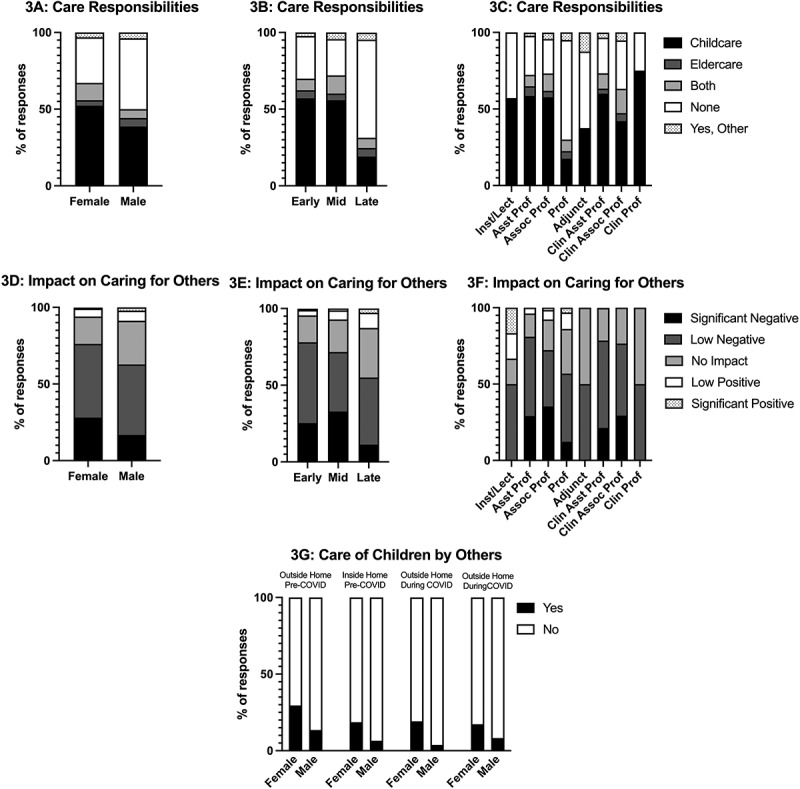
Responsibilities of caring for others (a-c, g) and the impact of pandemic on caring for others (d-f) within academic medicine faculty. Data separated by gender (a, d, g), career level (b, e), and rank (c, f).

The majority of respondents indicated that they were responsible for the care of someone else during the pandemic (e.g., children, elders; Figure 3). The responsibility of care by an individual was related to gender (χ2 (4) = 12.477, p = 0.014, φ = 0.198); 70% of female respondents indicated that they were involved in the care of someone else compared to 54% of males (Figure 3(a)). The need to care for others was also related to career level (χ2 (8) = 47.631, p < 0.001, φ = 0.275) and rank (χ2 (28) = 57.078, p = 0.001, φ = 0.214). Those at early- and mid-career levels were far more likely to be responsible for the care of others (Figure 3(b,c)). The impact felt by faculty secondary to caring for others during the pandemic was overall negative. While the impact on caring for others was not significantly related to gender (χ2 (4) = 8.527, p = 0.074, φ = 0.177), the respondent’s career stage and rank were (χ2 (8) = 18.378, p = 0.019, φ = 0.184 and χ2 (28) = 42.110, p = 0.042, φ = 0.197, respectively; Figure 3(d-f)). It was also influenced by degree type; those with a clinical degree were more likely to report negative impacts on caring for others compared to those with a non-clinical degree (χ2 (4) = 15.657, p = 0.004, φ = 0.238; 76% and 58% respectively). Relatedly, 52% of faculty reported a negative impact from increased household responsibilities. This impact was influenced by gender (χ2 (4) = 9.945, p = 0.041, φ = 0.179); 19% of women stated they were significantly negatively impacted compared to 9% of men. Rank (χ2 (28) = 52.086, p = 0.004, φ = 0.207) also significantly influenced this impact, as 19% of respondents in early- and mid-career levels were significantly negatively impacted by increased household responsibilities compared to only 4% of late career faculty.

How children were cared for did not change much from pre-pandemic to during the pandemic, except the percentage of those who utilized others to care for their children outside of the home, which decreased from 22% pre-pandemic to 11% during the pandemic (Figure 3(g)). There was a significant relationship between care of children by others both inside and outside of the home and gender, both pre-pandemic (inside the home: χ2 (1) = 10.444, p = 0.001, φ = 0.183; outside the home: χ2 (1) = 11.686, p = 0.001, φ = 0.194) and during the pandemic (inside the home: χ2 (1) = 5.621, p = 0.018, φ = 0.134; outside the home: χ2 (1) = 18.087, p < 0.001, φ = 0.241), where women respondents were more likely to have a child being cared for by others (Figure 3(g)).

Analysis of narrative responses from faculty outlined that childcare was a necessity to support working parents. Comments surrounded the need for *free onsite childcare* with flexible hours. Many respondents self-identified as not having children but saw the need for this support. Faculty commented on fearing bias and retribution for having to leave work or meetings to relieve childcare. Suggestions also included emergency childcare services, necessary for when faculty are required to be in-person but a daycare or secondary school is closed.

### The work life of academic medicine faculty has been overwhelmingly negatively affected by the pandemic

**Figure 4.**
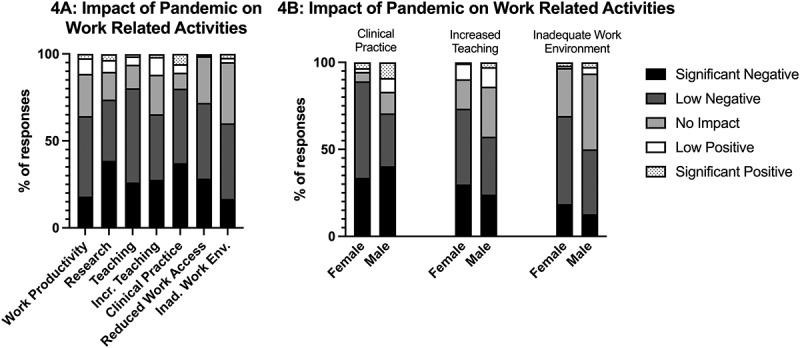
Impact of pandemic on work activities (a), separated by gender (b).

Many aspects of academic medicine faculty’s work were negatively impacted by the pandemic, including overall work productivity (64%), research (74%), teaching (80%), and clinical practice (79%; Figure 4(a)). We also saw secondary effects of the pandemic, such as increased teaching loads, reduced access to workspace, and inadequate work environments, also negatively impacted faculty (Figure 4(a)). Some of these effects were influenced by gender; females were more likely to report a negative impact on clinical practice (χ2 (4) = 15.013, p = 0.005, φ = 0.288), a negative impact of an increased teaching load (χ2 (4) = 9.917, p = 0.042, φ = 0.181), and a negative impact of an inadequate work environment (χ2 (4) = 12.646, p = 0.013, φ = 0.201; Figure 4(b)).

Career level and rank did not significantly influence these perceived impacts by faculty, however, degree type did. Those who hold a non-clinical degree perceived more significant negative impacts on research (48%), compared to 30% of clinical faculty. The perceived impact of not having access to their workspace was also influenced by degree type (χ2 (4) = 13.052, p = 0.011, φ = 0.204); those with a non-clinical degree experienced more significant negative impacts on their work by not having access to their workspace during the pandemic compared to those with a clinical degree (23%).

### Faculty provided actionable items to support them through and following the pandemic

While many faculty requested general support from institutions, frequently commented on were actionable items, including *safety measures, free onsite childcare* (reported above), *software and IT*, and *ease work burden*. Comments on *safety measures* highlighted the need for the institution to acknowledge faculty safety by providing appropriate PPE, COVID testing, vaccinations, and allowing faculty to continue to work from home.

Faculty comments requesting the institution to *ease work burden* noted the increased workload and the fact that as some faculty have taken a leave of absence, reduced hours, or left the workforce without being replaced, remaining faculty must carry the extra work. Respondents requested hiring faculty and staff to replace others and spread-out work to lift the increased burden fewer faculty are carrying. Comments to this end included: ‘*Accommodating people’s schedules is needed, but often falls on other co-workers to pick up the slack*.’; and ‘*Recognizing how many more hours that all the changes to our work require, and making some effort to decrease those hours back to nearer baseline*.’

The support subtheme *software and IT* was revealed as respondents commented on the need to equip themselves and their computers to adequately do their jobs at home. Of particular frustration was the need to deliver educational content in new ways without adequate software and training. Herein most faculty deemed professional development sessions inadequate and requested specific direction on how to prepare for remote learning.

### Institutions can play a role in helping faculty through the pandemic via compensation

No direct comments with respect to **compensation** were made requesting increase in salaries despite increased work hours, however comments highlighted not withholding annual cost of living and merit raises. While it was noted that the pandemic has had negative effects on institutional budgets, the subtheme *protected time/time off*, does not have direct costs associated. *Protected time/time off* can be granted by the institution to help faculty engage in scholarship, produce teaching materials, and participate in wellness activities.

*Compensation for home working environment* was revealed as respondents commented on the need to set up space at home to do their jobs. Reimbursement was requested for supplies, such as computers, increased internet service, and software necessary to work from home.

### The overall well-being of academic faculty has been negatively impacted, with early-career women being the most affected

**Figure 5.**
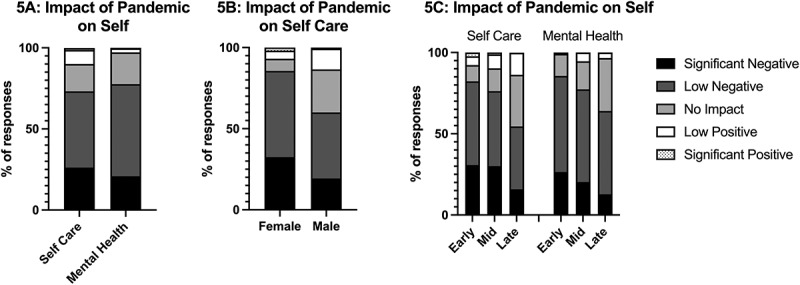
Impact of the pandemic on self-care and mental health in academic medicine faculty (a). The impact on self-care separated by gender (b), and the impact on self-care and mental health separated by career level (c).

The majority of respondents indicated a negative impact on their self-care (73%) and mental health (78%) during the pandemic (Figure 5(a)). These negative impacts have been influenced by multiple factors, including gender (χ2 (4) = 31.112, p < 0.001, φ = 0.314, self-care), career level (χ2 (8) = 28.584, p < 0.001, φ = 0.214 for self-care; χ2 (8) = 23.024, p = 0.003, φ = 0.314 for mental health), and degree type held (χ2 (4) = 10.818, p = 0.029, φ = 0.184, mental health).

Women were far more likely to report a negative impact on self-care compared to men (Figure 5(b)). Women also reported more negative impacts on mental health (82%) compared to men (73%), however this was not significantly different. While rank did not have a significant impact on self-care and mental health, we found those early in their careers had far more negative impacts on both self-care and mental health than their more senior colleagues (Figure 5(c)). We also found that those who had a clinical degree were twice as likely (27%) to indicate a significant negative impact on their mental health compared to those who did not hold a clinical degree (14%).

### Faculty identified ways institutions can help them with their personal and mental health

A critical way the institution can help faculty is in the theme of mental health. Many comments highlighted the need for increased access to mental health resources, noting inadequate suggestions to ‘*sleep more, eat better, be social, take this additional class …* ’ when there is no time to do so. Respondents noted providing *access to a behavioral health professional* to be beneficial, as existing behavioral health faculty are being overburdened by their colleagues’ requests for help, and many faculty are unequipped with skills to support themselves, their families, their students, and their mentees.

Another subtheme of mental health is to *be understanding and empathetic*. This was highlighted as creating outlets for discussion, as well as assessing mental health: ‘*It would be nice for them to assess the employees mental wellbeing more hands on than in a news article about a wellness corner*.’ This dovetails into the *inequality stress* subtheme that emerged from comments about unequal workload, not receiving the same considerations that students receive, and a multitude of requests from respondents on what institutions should stop doing: ‘*Stop piling on more and more expectations*’; ‘*Stop blaming faculty for the institution’s financial difficulties*’; ‘*Stop telling faculty to take care of ourselves*’; and ‘*Stop telling faculty to turn off at night and weekends*’. Respondents attribute the increased stress and decline in mental health to the extra work expectations that make it impossible to take care of themselves and turn off after hours, creating a vicious cycle. This *inequality stress* subtheme also has a inverse relationship to the subthemes *ease work burden* under support and *protected time/time off* under compensation, as the increase in protected time and ease of work burden would help to relieve the stress.

### Early-career women in academic medicine feared retribution for taking a leave of absence or tenure clock extension and had more concerns about future job promotions

Neither rank, career level, nor gender influenced whether one considered taking a leave of absence (LOA) or requested a tenure-clock extension. However, 38% of women indicated that they feared institutional retribution if they were to take a leave of absence or request a tenure clock extension compared to 23% of men (χ2 (1) = 4.945, p = 0.026, φ = 0.160). Similarly, only 61% of women indicated that they felt the pandemic would not negatively impact future job promotions compared to 74% of men (χ2 (2) = 6.315, p = 0.043, φ = 0.141). Those earlier in their career (43%) were more likely to fear retribution (χ2 (2) = 8.960, p = 0.011, φ = 0.216) compared to those in mid- (29%) or late career stages (32%). Those early in their career (32%) were also more likely to have concerns that the pandemic would impact their future job promotions (χ2 (4) = 14.542, = 0.006, φ = 0.152p) compared to mid- (24%) and late career (11%) individuals. Faculty commented that if they knew there would not be a possibility of retribution, they would consider taking a LOA or tenure clock extension.

### Faculty expressed a desire for flexibility in work expectations to address their inability to achieve work promotion metrics due to the pandemic

The theme flexible expectations stemmed from comments about the inability to achieve promotion metrics as a result of changes in productivity. The subthemes that emerged include *grant funding, tenure clock pause & extension, publication numbers, credit for all extra contributions*, and *reduction of expectations*. Under the subtheme *grant funding*, respondents highlighted the need to relax grant funding expectations as little research progress has been achieved during various stages of shutdown and to provide bridge funding to help junior faculty whose funding has been interrupted.

The *tenure clock pause & extension* subtheme was mixed, as faculty want the ability to pause or add time to their tenure clock, but with the caveat that it would not have a negative connotation. This could be addressed by suggestions made under the *reduction of expectations* subtheme, as respondents commented on the need to be more lenient on all faculty productivity expectations, including publication numbers, grants awarded, teaching approaches, committee expectations, and mentoring. This calls for an overhaul of expectations: ‘*There needs to be a fundamental reappraisal of job expectations, teaching approaches, committee expectations, etc … I could spend all my time on Zoom, 8 am – 9 pm some days and weekends and it is just not sustainable*.’

The reappraisal of job expectations could include giving promotional *credit for extra contributions*, as faculty have been forced to contribute to the workforce in time consuming ways that do not yield academic products.

### Increased and better communication were highlighted as important to faculty

The communication theme describes ways institutional communication can be used to help faculty, including a *general increase, include faculty in the discussion, acknowledge extra efforts*, and *create an action plan*. Many faculty lamented that institutions have been using broad, generalized statements to communicate, which can be disengaging. Comments on this highlight the need to increase communication, update websites, and include more specific information for subsets of faculty, staff, and students.

Under the subtheme of *include faculty in the discussion*, respondents highlighted the disconnect between the administration making decisions affecting faculty without considering the ramifications of those decisions. Comments clearly show that decisions and changes have been made without consulting the faculty who have to implement them: ‘*Additionally include faculty in the decision making processes so that all the ramifications of decisions are known upfront rather than the chaotic cascade that follows another new mandate without consideration for the teaching faculty*.’ Including faculty in these communications could reduce this negative cascade ‘*Change leadership. Allow the people who are on the ground to affect positive change*.’

Faculty highlighted a seemingly simple, yet unaddressed, subtheme in *acknowledge extra efforts*. Institutions can positively impact faculty by communicating respect and recognition for sacrifices faculty have made for their jobs. A way for institutions to provide stability for faculty is to *create an action plan*. Many comments surround the constant change or vague narrative provided by institutions, including how the pandemic is negatively affecting people, without communicating an action plan. Clear communication of action plans, including amended promotion and tenure expectations and how to mediate the perceived negative effects, were highlighted by respondents as ways to help faculty being negatively affected.

## Discussion

This study details the effects of the COVID-19 pandemic on academic faculty and elucidated the inequities in academic medicine as a result of the pandemic. To the authors’ knowledge, this is the first study to formally evaluate the impact of the pandemic on academic medicine faculty, supported by thematic analysis of narrative comments. It is clear that inequities existed prior to the pandemic, and the results of this study show that in just 12 months, the inequities women experience in academic medicine were exacerbated. Research, teaching, and clinical practice were negatively impacted for all faculty in academic medicine, more so by women. Women and early career faculty were also more negatively impacted and were more likely to be responsible for caring for others. Women, early career, and clinical faculty were more likely to experience negative impacts on self-care and mental health. It is clear that faculty are experiencing increased workloads, some forced to complete them in an inadequate work environment, while having increased demands at home, and feeling the fallout in the areas of self-care and mental health. To make matters worse, early career women were more likely to have concerns that the pandemic would impact future job promotions and fear institutional retribution if they were to request a leave of absence or a tenure clock extension. These data contribute to the opinion pieces that have outlined an increased impact of the pandemic on women and support those that are sounding alarms that women in academic medicine are facing a crisis [28, 31, 35–39], as well as supports data recently published by the NIH that early-career faculty are more concerned about the impact of the pandemic on their career trajectory [46].

Childcare was a well-known pre-pandemic hurdle for women in academic medicine [8,47], and the pandemic only heightened the difficulties for women with children to be productive in the workforce. Many primary schools and centers continue today in a hybrid format with decreased hours of supervised care for children. The present study found that women were more likely to be responsible for childcare following the closure of these schools due to the pandemic. Overall, faculty felt negatively impacted by these increased childcare responsibilities, as predicted by Robinson and colleagues [31] Childcare can be cost prohibitive, which may explain the significant negative impact we found early career faculty felt compared to other career levels, and it is not offered at night and on weekends, which also may explain the significant negative impact we found clinical faculty felt compared to non-clinical faculty. Additionally, clinical shifts increased as a result of the overburdened healthcare system at the onset of the pandemic and continue to be full as health-care workers see patients who may have put off care throughout the pandemic. These increased hours with little flexibility as compared to basic science faculty likely contribute to the significant negative impact on clinical faculty. One way respondents in the present study outlined to address this negative impact is to provide onsite childcare, which is not common outside of undergraduate settings and uncommonly offered to academic medicine faculty, and also noted the need for free childcare with extended hours and spots available for emergencies. Faculty also related the need for childcare to fear of bias and retribution, as they report colleagues without similar responsibilities view them as less productive because they have childcare responsibilities. If an institution wants to make an impact on the pipeline effect and help elevate early career women, providing childcare options is imperative.

Although there were no gender differences in whether one considered taking a LOA or tenure clock extension, women were more likely to indicate that they feared institutional retribution for doing so. This fear is not unsubstantiated, as previous work has highlighted the inequities in parental pauses, as men are able to be more productive and women are tied to being caretakers [48]. Fear of retribution is also tied to themes of institutional flexible expectations, support, communication, and mental health, as requests were made to reduce promotion expectations, spread work across faculty, promote a team approach to reduce stress, and involve faculty being affected in creating an action plan. If faculty were more involved in the communication, perhaps the fear of retribution we saw in the current study would be eased.

As pre-pandemic compensation gaps were wide [15], post-pandemic gaps are likely to be wider. While the present study did not directly assess compensation, the data demonstrate the differential impact that the pandemic has had on women. Compensation did arise out of narrative comments, and not at the request to be paid more, but conversely to be paid *fairly*. As institutions have had to reallocate budgets to PPE, testing, and acquiring software to make remote education and patient care possible, faculty salaries were frozen and those in administrative leadership positions with substantially larger salaries were cut. Early career and women faculty were already at a disadvantage, and with no merit or cost of living increases for two fiscal years at many institutions, these faculty are rapidly losing ground. Further, loss of discretionary funding that is important for attending virtual professional meetings and networking, forces faculty to pay out of pocket for these experiences that are imperative for up-to-date practice, research, and promotion. Although institutions are not actively cutting salaries of faculty at lower ranks, their lack of compensation and support for these faculty have ripple effects that may put women in academic medicine decades behind.

The parallel between those who occupy the advanced ranks and leadership positions and who are least impacted by the pandemic needs to be emphasized. The results show that early career faculty were more likely to be negatively impacted by the pandemic, and the extra work and expenses incurred in attempts to make up for inadequate work environments experienced by early career faculty, who are already identified as being inadequately compensated, created unequal stress. Yet it is late career faculty, who are predominantly men, that are least affected and making the decisions to cut funding, increase teaching loads, increase patient loads, and decrease office space, all of which ultimately compound these negative impacts. As the communication theme and subsequent subthemes describe, it is imperative that faculty directly affected by the decisions made by administration are included in the discussion and action plan creation.

The attention to supporting patients and students has been great and expected as they are deemed the consumers. Many resources have been used to ensure adequate care, safety, and education [49–51]; however, little attention has been focused on the individuals providing these services [31,52,53], as is supported by the majority of our respondents. The demands on faculty increased to astronomical levels throughout the pandemic and have not let up, yet there has been little work to balance the unequal efforts faculty have made. Clinical faculty faced high patient mortality rates with little time to adjust to the new methods of treating patients, constantly working to learn about an unknown disease while also serving as their patient’s last goodbyes. The need for easy access to mental health resources, without the stigma for utilizing them, is paramount. While some faculty request simple recognition for these efforts, most are asking for a break in the form of leniency in promotion requirements, time off, spreading out the workload, reduction of unnecessary meetings, and placing restrictions on scheduling meetings outside normal work hours. These low-cost options have high rewards in the form of lowering faculty stress and helping work-life balance, both of which would benefit faculty mental health.

Limitations of this study include the relatively small sample size and low representation of faculty in different regions of the country, however responses are likely representative of the majority of faculty in academic medicine. The goal was to gather numerous responses from faculty across the country, however survey fatigue may play a factor in the limited number of completed questionnaires. This study focused on academic medicine faculty within the U.S. as the systemic support for family leave and childcare is superior in other countries. However, this can serve as a template for similar studies with a larger reach in locations outside of the U.S. This was a cross-sectional study providing self-reported data and can be further expanded upon through future longitudinal employment, promotion, and productivity studies. Further, a pilot test of the questionnaire was unable to be completed, therefore literature and local experts were used in its construction. Validity to results was further added through strong quantitative and qualitative methodology. The intention is this study will provide a baseline for larger studies in academic medicine.

Pre-pandemic stress and burnout were widely prevalent among faculty in US medical schools [15], and a year into the pandemic, as the world slowly is reopening and rejoicing the advent of vaccines, the data clearly show that academic medicine faculty are still suffering. The fallout from this may last years beyond the general population as administrations continue to concentrate on patient care and student education above the health, wellness, and education of their employees. The effects of the pandemic on academic medicine faculty have been outlined in the present study with the intention to bring about awareness and influence institutional change. The responses from academic medicine colleagues are harrowing and require immediate attention, particularly for women. Administrations must recognize and validate the glass ceiling that has thickened for women in academic medicine and make substantial changes.
